# Public health diplomacy in Central Asia: a scoping review

**DOI:** 10.3389/fpubh.2026.1926850

**Published:** 2026-09-16

**Authors:** Ashish Joshi, Nishtha Sharma, Niharika Jha, Jonas Cruz, Nurzhan Mukashev, Khuseyn Nazarzoda, Sukhrob Saliev, Ainash A. Sharshenova, Antonio Sarria-Santamera

**Affiliations:** 1 School of Public Health, University of Memphis, Memphis, TN, United States; 2 Department of Medicine, School of Medicine, Nazarbayev University, Astana, Kazakhstan; 3 Center for Health, Policy and Management, School of Social Sciences, Business and Law, SDU University, Kaskelen, Kazakhstan; 4 Department of Environmental Health, School of Public Health, Avicenna Tajik State, Medical University, Dushanbe, Tajikistan; 5 Westminster International University in Tashkent, Tashkent, Uzbekistan; 6 Department of Public Health, International Higher School of Medicine, Bishkek, Kyrgyzstan; 7 Department of Biomedical Sciences, School of Medicine, Nazarbayev University, Astana, Kazakhstan

**Keywords:** Central Asia, cross-border cooperation, health governance, health system reform, public health diplomacy, scoping review

## Abstract

**Background:**

Central Asia comprising Kazakhstan, Kyrgyzstan, Tajikistan, Turkmenistan, and Uzbekistan, confronts a complex array of health challenges shaped by the post-Soviet transition, including fragmented health systems, a dual burden of communicable and non-communicable diseases, environmental degradation, healthcare inequality, and competing geopolitical influences. Public health diplomacy has emerged as a critical instrument for addressing these transnational challenges.

**Objectives:**

This scoping review aimed to map and synthesize the existing evidence to examine the role of public health diplomacy in strengthening health governance and regional and international health cooperation.

**Methods:**

Following the Arksey and O’Malley scoping review framework, this paper used a systematic search of PubMed, and Google Scholar. Studies published between 2000 and 2025 addressing health diplomacy, health governance, cross-border cooperation, or health system reform in Central Asia were included. Data was extracted using a standardized charting form and synthesized through descriptive and narrative thematic analysis.

**Results:**

Seventeen studies met the inclusion criteria. Kazakhstan was the most extensively studied country (*n* = 4), followed by Uzbekistan (*n* = 2), Kyrgyzstan (*n* = 1), and Tajikistan (*n* = 1). No country-specific study addressed Turkmenistan. Key findings revealed: (i) all five countries continue to navigate the post-Soviet health transition with uneven progress toward UHC; (ii) significant intra-national healthcare inequalities persist, particularly in Kazakhstan’s rural areas; (iii) environmental health threats including transboundary water pollution, industrial contamination, and household air pollution, demand coordinated diplomatic responses; (iv) the European Union uses health as a soft-power instrument, while China’s Belt and Road Initiative and the SCO represent expanding but under-evaluated platforms for health cooperation; (v) Russia’s influence persists through institutional legacies and migrant health governance; (vi) centralized, top-down health communication hinders public trust across the region; and (vii) no health diplomacy training program exists for Central Asia. The strongest SDG 3 alignment was with target 3.8 UHC, while maternal mortality, child health, and substance abuse targets remained unexamined through a PHD lens.

**Conclusion:**

Public health diplomacy has significant potential to strengthen health governance and regional cooperation in Central Asia. Advancing this agenda requires stronger regional collaboration, context-specific capacity building, greater evidence generation, and systematic evaluation.

## 1 Background

Central Asia: Kazakhstan, Kyrgyzstan, Tajikistan, Turkmenistan, and Uzbekistan; is home to 78 million people at the crossroads of Europe and Asia (Table 1). All five republics inherited the Soviet Semashko health model, a state-funded, centralized system providing universal free access through extensive hospital networks and salaried physicians, with no private healthcare provision (1, 2), which collapsed after 1991, triggering life expectancy declines, infrastructure deterioration, and a dual disease burden (2, 3).

**Table 1:** Demographic, economic, and health system indicators for Central Asian countries.

Indicator	Kazakhstan	Kyrgyzstan	Tajikistan	Turkmenistan	Uzbekistan
Population (2024)	20,592,571	7,221,868	10,590,927	7,494,498	36,361,859
GDP per capita, US$ (2024)	$14,154.60	$2,420.20	$1,341.20	$6,856.70	$3,161.70
World Bank Income Classification	Upper-middle	Lower-middle	Lower-middle	Upper-middle	Lower-middle
Life Expectancy at Birth, years (2024)	75	72	72	70	73
Health Expenditure per Capita, US$ (2023)	$489.71	$96.65	$85.15	$581.38	$192.12
UHC Service Coverage Index (2023)	83	74	72	81	79
Out-of-Pocket Expenditure (% of health spending)	27.5% (2020)	46.3%*	63.5%*	75.6% (2022)	53.1% (2020)
Physicians per 10,000 population	40.0 (2020)	22.0 (2021)	17.0 (2020)	22.2 (2014)	26.0 (2021)
Health Insurance Coverage	Mandatory Social Health Insurance (SHI) (2020)	69% Mandatory Health Insurance Fund (MHIF)	No universal scheme	State-funded (limited data)	State-funded + private emerging
Key Health System Reform	Social health insurance; primary care gatekeeping	Family medicine model; single-payer MHIF	Basic benefits package (ended 2023); UHC by 2030 commitment	Centralized state model; limited reform transparency	Post-2016 opening; expanded WHO engagement
Health Governance Model	Decentralized; purchaser-provider split	Semi-decentralized; MHIF as purchaser	Centralized; Ministry of Health (MOH) dominant	Highly centralized; state monopoly	Centralizing; MOH-led reform
Pharmaceutical Regulatory Framework	EAEU harmonized; national drug formulary	EAEU harmonized; essential medicines list	National regulation; WHO prequalification reliance	State-controlled procurement; limited data	National agency; expanding WHO alignment
Key Public Health Challenges	Urban–rural inequality; Non Communicable Diseases (NCDs); environmental pollution; declining rural facilities	High OOP spending; workforce maldistribution; COVID-19 vulnerabilities	lowest per-capita health expenditure; physician shortage; cross-border TB; discontinued benefits	Data gap; limited transparency; Limited publicly available health data	TB care transition; (Water, Sanitation, and Hygiene)WASH-related diseases; Aral Sea legacy

Data represent the most recent publicly available year identified for each indicator and may therefore vary across countries and indicators. Sources include: World Bank World Development Indicators (2024), retrieved from https://data.worldbank.org; WHO Global Health Observatory, UHC Service Coverage Index (2023), retrieved from https://data.who.int/indicators/i/3805B1E/9A706FD. SHI, Social Health Insurance; MHIF, Mandatory Health Insurance Fund; EAEU, Eurasian Economic Union.

Each country has pursued distinct reforms: Kazakhstan introduced social health insurance, Tajikistan committed to Universal Health Coverage (UHC) by 2030, Uzbekistan expanded international engagement after 2016, yet all face persistent challenges in financing, rural access, and equitable service delivery. Central Asia sits at the convergence of competing geopolitical influences. Russia maintains ties through the Commonwealth of Independent States (CIS) and Eurasian Economic Union (EAEU), with millions of labor migrants creating health governance needs. China’s Belt and Road Initiative and Health Silk Road expand health infrastructure through the Shanghai Cooperation Organization (SCO). The European Union (EU) deploys health as soft power (4), while World Health Organization (WHO), the World Bank, and United Nations Development Program (UNDP) provide technical assistance. This multipolar environment creates diverse opportunities but risks fragmenting cooperation when diplomatic interests diverge from health needs.

Health diplomacy (HD) in Central Asia is a vital mechanism for regional cooperation, linking public health infrastructure with geopolitical strategy. The evolution of public health diplomacy (PHD) in the region has occurred alongside major political transitions and growing regional and international engagement, from the post-Soviet transition to contemporary multilateral health partnerships (Figure 1). These developments illustrate how political and diplomatic processes have increasingly created mechanisms for health system reform, regional health security, capacity building, and cross-border cooperation. Health diplomacy refers to the negotiation and governance processes through which actors address health challenges across national borders. Katz et al. (5) formalized this concept at the intersection of public health, international affairs, law, and economics. Kickbusch et al. (6) then introduced global health diplomacy (GHD), arguing that health’s embeddedness in foreign policy demanded new strategic approaches for multilateral negotiations. More recently, Joshi et al. (7) defined public health diplomacy (PHD) as “a multidisciplinary field that enables its practitioners to effectively communicate, facilitate, negotiate, and build consensus using systems thinking, evidence-based, community-informed approaches, grounded in equity-focused and human-centered values to improve health and well-being for all.”

**Figure 1:**
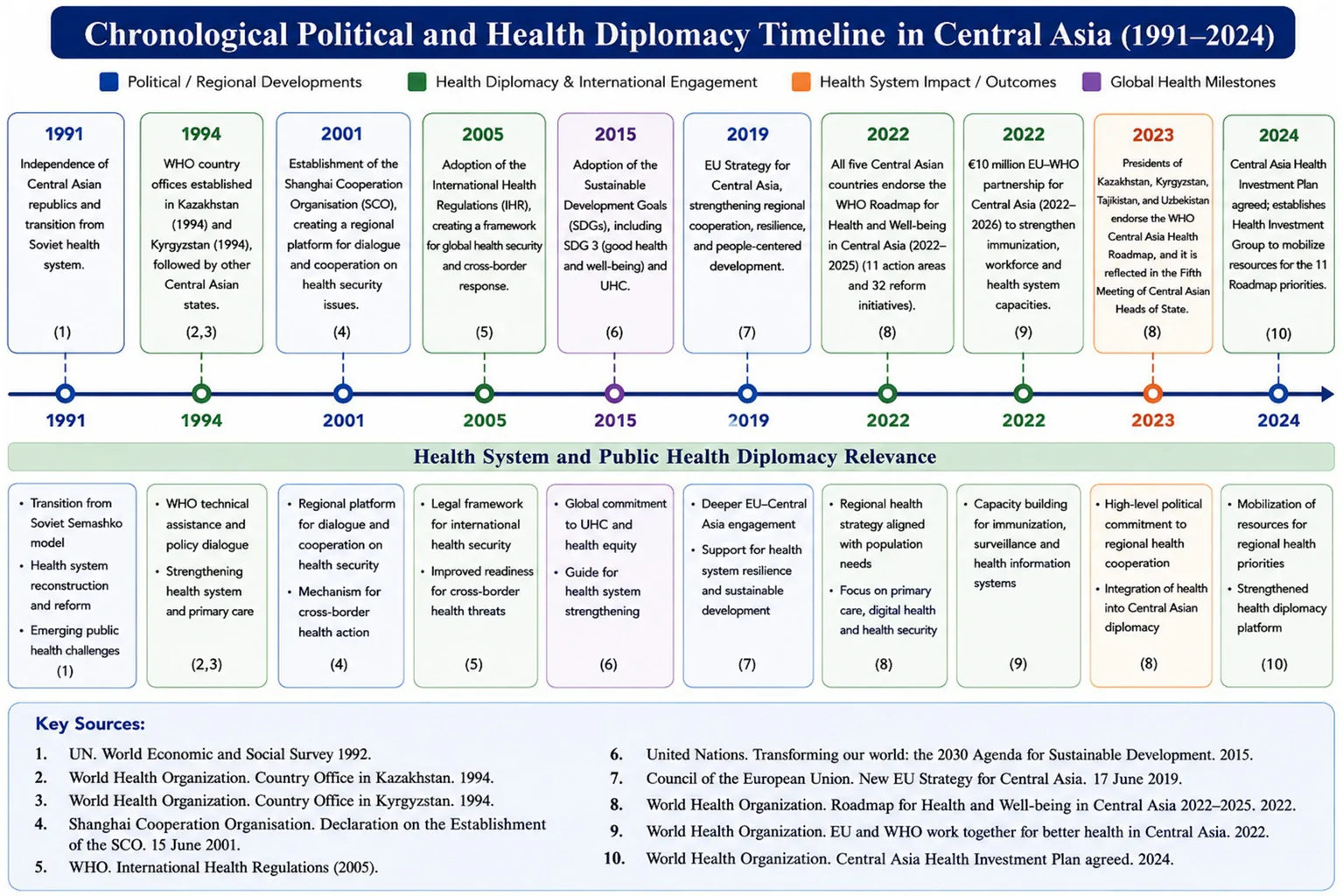
Chronological timeline of key political and health diplomacy developments influencing health systems in Central Asia, 1991–2024.

PHD is a new paradigm in global health perspective and a new chapter for Central Asian region in terms of a discipline. PHD can be employed to transform Central Asia’s health landscape through five critical roles. First, PHD strengthens health systems by facilitating cross-country policy learning, enabling nations to share reform experiences rather than navigating transitions in isolation. Second, PHD addresses transnational threat like transboundary water pollution, the Aral Sea legacy, cross-border tuberculosis, pandemic preparedness, by building coordinated surveillance and governance across borders. Third, PHD empowers Central Asian professionals to negotiate effectively within the SCO, CIS, WHO, and World Bank, ensuring regional health priorities are represented rather than externally imposed. Fourth, PHD reduces healthcare inequality and catastrophic spending by promoting evidence-based financing reforms and coordinating donor investments. Fifth, PHD builds public trust by fostering transparent, community-informed communication that replaces centralized Soviet-era models.

Public health diplomacy can influence health systems by facilitating policy dialog, mobilizing financial and technical resources, strengthening cross-border cooperation, and supporting the transfer of knowledge and institutional capacity. In Central Asia, these mechanisms are particularly relevant because health system development has occurred alongside post-Soviet political transitions and growing engagement with regional and international actors. Examining these interactions provides an opportunity to understand public health diplomacy not only as a foreign policy mechanism but also as a potential instrument for health system strengthening and regional health governance.

Despite the growing importance of public health diplomacy in addressing transnational health challenges, no previous review has comprehensively mapped the evidence on its role in shaping and strengthening the health landscape of Central Asia. To address this gap, this scoping review aims to: (i) examine country-specific health governance systems across the five Central Asian republics; (ii) map the existing literature on health diplomacy and public health diplomacy in the region; (iii) analyze international and regional public health diplomacy mechanisms that influence health cooperation; (iv) assess the alignment of health diplomacy initiatives with Sustainable Development Goal 3 (SDG 3) and Universal Health Coverage (UHC); and (v) identify geographic, thematic, and methodological gaps in the current evidence base on health governance and public health diplomacy in Central Asia. The Central Asian countries included in this review are Kazakhstan, Kyrgyzstan, Tajikistan, Turkmenistan, and Uzbekistan.

## 2 Methodology

This scoping review follows the methodological framework proposed by Arksey and O’Malley (8), which provides a structured, transparent, and replicable approach to mapping the breadth of existing evidence on a given topic. This framework was selected for its suitability to explore emerging and interdisciplinary fields where the evidence base is diverse and heterogeneous. The review was conducted in accordance with the Preferred Reporting Items for Systematic Reviews and Meta-Analyses Extension for Scoping Reviews (PRISMA-ScR) guidelines to ensure rigor in reporting. The research team conducted the literature search and screening process between March 2026 and April 2026.

### 2.1 Identifying the research question

The following research question guided this review: What evidence exists regarding the current landscape of health diplomacy in Central Asia, and how can public health diplomacy strengthen health governance, enhance cross-border health cooperation, support policy development and capacity-building initiatives, and advance progress toward Sustainable Development Goal 3 (SDG 3) and Universal Health Coverage (UHC)?

### 2.2 Identifying relevant studies

The research team conducted a systematic search to identify studies related to health diplomacy in Central Asia, with eligibility criteria defined *a priori* to ensure consistency and transparency in study selection. The information sources for this review included the following electronic databases: PubMed, and Google Scholar, to capture peer-reviewed biomedical, social science, and health policy literature. Additionally, Google was used to ensure coverage of gray literature, policy reports, institutional publications, and less traditional academic outputs (e.g., WHO country reports, UNDP assessments, regional health cooperation agreements, and training manuals).

The team conducted targeted searches using combinations of key terms. The search strategy employed Boolean operators (AND, OR) to combine the following groups of terms presented in Table 2. Studies were eligible for inclusion if they comprised peer-reviewed articles, conference papers, reports, policy documents, or relevant gray literature addressing health diplomacy, public health diplomacy, health governance, cross-border health cooperation, or bilateral and multilateral health agreements involving one or more Central Asian countries. Eligible publications also included studies examining health diplomacy competencies, training frameworks, capacity-building initiatives, and the intersection of public health, foreign policy, and international relations in the region, as well as documents assessing the role of international organizations such as the World Health Organization, United Nations Development Program, World Bank, and Shanghai Cooperation Organization in shaping regional health diplomacy. The review included studies published between 2000 and 2025 in English or Russian. Studies were excluded if they were unrelated to health diplomacy, public health governance, or cross-border health cooperation in Central Asia; focused exclusively on clinical or biomedical issues without a policy, governance, or diplomatic component; presented theoretical or conceptual discussions without reference to specific initiatives, programs, policies, or country-level evidence from the region; consisted solely of editorials, commentaries, or opinion pieces lacking empirical or documentary evidence; or focused on other geographic regions without substantial relevance to Central Asia.

**Table 2:** Search strategy terms.

Concept group	Search terms
Health Diplomacy	“Public health diplomacy” OR “global health diplomacy” OR “health diplomacy” OR “health governance” OR “health cooperation” OR “health foreign policy”
Central Asia	“Central Asia” OR “Kazakhstan” OR “Kyrgyzstan” OR “Tajikistan” OR “Turkmenistan” OR “Uzbekistan”
Health Systems and Policy	“Health systems” OR “universal health coverage” OR “UHC” OR “SDG 3” OR “health policy” OR “health reform”
Diplomacy and Cooperation	“Bilateral cooperation” OR “multilateral cooperation” OR “cross-border health” OR “regional cooperation” OR “international health regulations” OR “health security”
Organizations	“WHO” OR “World Bank” OR “UNDP” OR “Shanghai Cooperation Organization” OR “SCO” OR “Commonwealth of Independent States” OR “CIS”

### 2.3 Study selection

The research team imported all retrieved citations into reference management software (Zotero). Following de-duplication, two reviewers independently screened titles and abstracts for relevance against the predefined inclusion and exclusion criteria. Articles passing the initial screening underwent full-text review. Any discrepancies between reviewers were resolved through discussion and consensus, with a third reviewer consulted where necessary. The PRISMA flow diagram (Figure 1) reflects the complete study selection process, including the number of records identified, screened, excluded (with reasons), and included in the final review.

### 2.4 Charting the data

The team developed a standardized data charting form in Microsoft Excel to ensure consistency in extracting and organizing information from the included studies. The charting process captured key characteristics of each publication, as outlined in the data extraction framework (Table 3).

**Table 3:** Data extraction framework.

Data field	Description
Study ID	Unique identifier assigned to each included study
Author(s) and Year	Lead and co-authors, year of publication
Country/Region Focus	Central Asian country or countries addressed
Study Design/Type	Peer-reviewed article, report, policy document, gray literature
Type of Health Diplomacy Initiative	Bilateral/multilateral agreement, health governance reform, cross-border program, training initiative, policy framework
Key Actors/Stakeholders	Government agencies, international organizations (WHO, World Bank, UNDP, SCO, CIS), NGOs, academia, private sector
Health Domain/Topic	Communicable diseases, NCDs, maternal and child health, health security, UHC, health systems strengthening
Alignment with SDG 3/UHC	Evidence of explicit linkage to SDG 3 targets or UHC service coverage indicators
Key Findings/Outcomes	Main results, policy recommendations, capacity-building outcomes, diplomatic achievements
Gaps/Limitations Identified	Challenges reported, evidence gaps, structural barriers to health diplomacy

### 2.5 Collating, summarizing, and reporting the results

The extracted data were synthesized using a combination of descriptive statistics and narrative synthesis. Frequency and distributions were calculated to provide an overview of the types of health diplomacy initiatives, geographic focus, key actors involved, health domains addressed, and alignment with SDG 3 and UHC indicators across the included studies. Health diplomacy initiatives were mapped by Central Asian countries, thematic area (e.g., communicable disease control, health systems strengthening, health security, maternal and child health), and type of diplomatic engagement (bilateral, multilateral, regional). This mapping allowed the identification of patterns, gaps, and contextual variations in public health diplomacy across the region.

In addition, a qualitative thematic analysis was conducted to identify recurring themes, key challenges, and emerging insights across literature. The analysis explored the extent to which public health diplomacy in Central Asia has addressed regional health priorities, facilitated cross-border cooperation, and contributed to progress toward SDG 3 and UHC. To enhance clarity and accessibility, findings are presented using summary tables and thematic charts that visually map the distribution of initiatives across countries, health domains, and diplomatic frameworks. The results are structured around the following thematic areas: (i) geographic distribution of initiatives; (ii) types of health diplomacy engagements; (iii) key actors and stakeholders; (iv) health domains and policy priorities; (v) alignment with SDG 3 and UHC; and (vi) gaps and recommendations.

To complement the evidence identified through the scoping review and provide context for cross-country comparisons, publicly available data were obtained from authoritative international sources, including the World Health Organization (WHO), World Bank, United Nations (UN), and relevant regional and multilateral organizations. These contextual data included selected demographic, economic, health financing, health workforce, Universal Health Coverage (UHC), and health system indicators, as well as information on major political and health diplomacy milestones in Central Asia. These sources were used solely to contextualize and support interpretation of the review findings and were not considered part of the formal scoping review search, screening, or study selection process.

### 2.6 Ethical considerations

As this study is a scoping review of publicly available literature and does not involve primary data collection from human participants, ethical approval was not required. All data were sourced from published peer-reviewed articles, institutional reports, and publicly accessible policy documents.

## 3 Results

A total of 17 studies met the inclusion criteria and were included in this scoping review (Figure 2). These studies, published between 2000 and 2025, encompassed health system reviews, scoping reviews, cross-sectional studies, qualitative investigations, policy analyses, and commentary papers. The included literature addressed multiple dimensions of public health diplomacy (PHD) in Central Asia, including health system governance, cross-border cooperation, universal health coverage (UHC), environmental health diplomacy, disease surveillance, health communication, and the role of international organizations in shaping health agendas across the five Central Asian republics: Kazakhstan, Kyrgyzstan, Tajikistan, Turkmenistan, and Uzbekistan.

**Figure 2:**
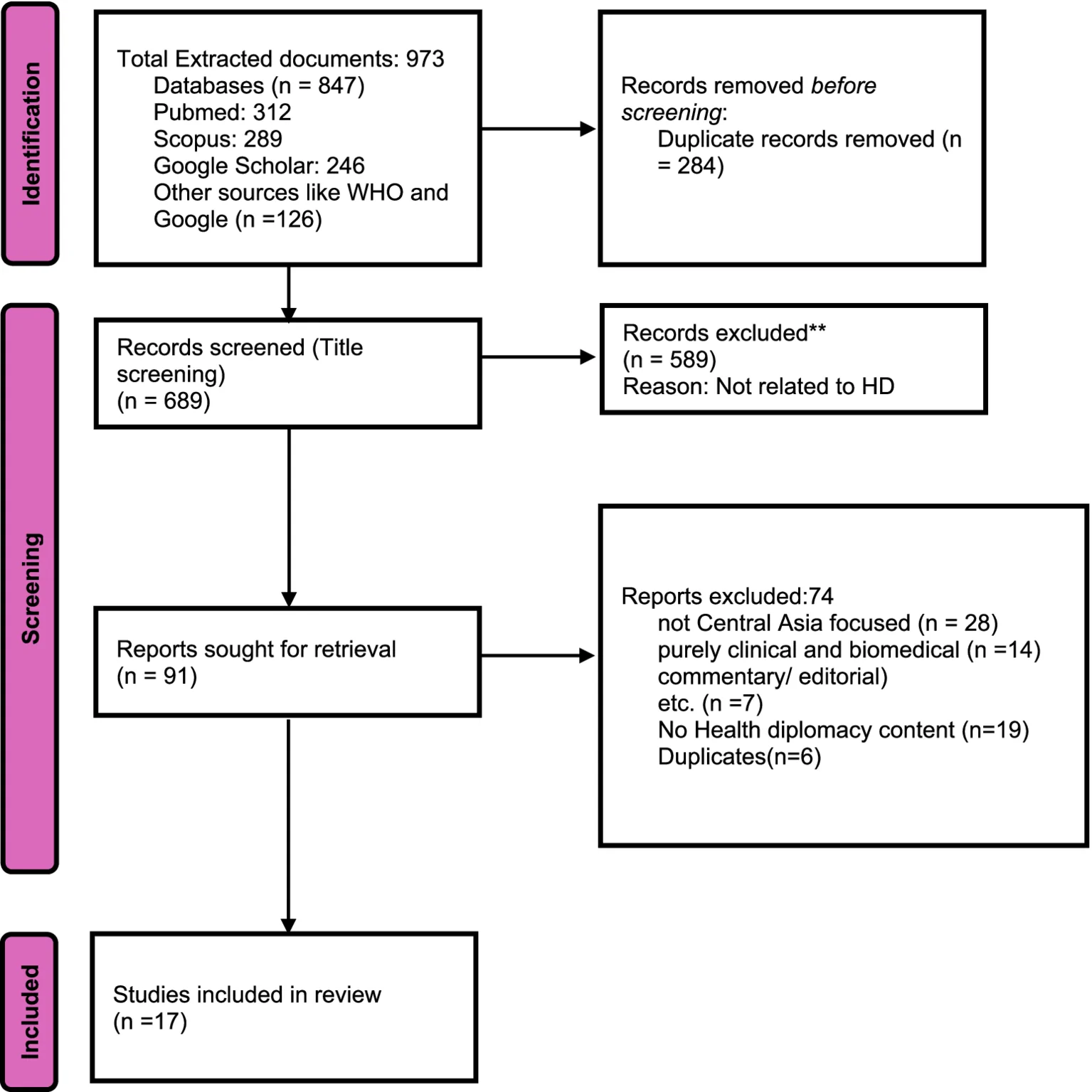
PRISMA flow diagram: Study identification, screening, and inclusion process.

The results are organized into four major sections. First, we present an overview of the included studies. Second, we examine health diplomacy within each Central Asian country, analyzing country-specific health governance, health system reform, and diplomatic engagement. Third, we examine international health diplomacy with Central Asia, including bilateral, multilateral, and institutional cooperation. Forth, we present an assessment of gaps and cross-cutting themes that emerged from the synthesis. Finally, we linked the existing health diplomacy literature to show how Public Health Diplomacy can be incorporated with these gaps and cross-cutting themes that emerged from the synthesis. Summary of Findings are presented in Table 4.

**Table 4:** Summary of included studies (*n* = 17).

#	Authors (Year)	Title	Journal/Source	Country focus	DOI
1	Adambekov et al. (3)	Health challenges in Kazakhstan and Central Asia	J Epidemiol Community Health, 70(1), 104–108	Central Asia	10.1136/jech-2015-206251
2	Askarov et al. (36)	Health status and quality of life near Zhezkazgan copper smelter	J Environ Public Health, 2023, 8,477,964	Kazakhstan	10.1155/2023/8477964
3	Baspakova et al. (37)	Socioeconomic and demographic factors influencing dietary patterns in Aktobe	Front Public Health, 13, 1,687,222	Kazakhstan	10.3389/fpubh.2025.1687222
4	Chattu et al. (34)	Access to medicines through global health diplomacy	Health Promot Perspect, 13(1), 40–46	Global	10.34172/hpp.2023.05
5	Collins et al. (4)	Negotiated health diplomacy: EU and Central Asia	Hague J Diplomacy, 13(4), 432–456	Central Asia	10.1163/1871191X-14010032
6	Guo et al. (17)	Transboundary water and energy problems in Central Asia	SpringerPlus, 5(1)	Central Asia	10.1186/s40064-016-3616-0
7	Jobalayeva et al. (35)	Rural healthcare network in Kazakhstan	Sci Rep, 15	Kazakhstan	10.1038/s41598-025-11432-w
8	Kickbusch & Liu (27)	Global health diplomacy—Reconstructing power and governance	The Lancet	Global	10.1016/S0140-6736(22)00583-9
9	Kohler et al. (10)	Health system support and strengthening: TB treatment in Uzbekistan	Health Econ Rev., 6(1), 28	Uzbekistan	10.1186/s13561-016-0100-z
10	Kyrgyzstan HSR (2023)	Kyrgyzstan: Health System Review	WHO European Observatory	Kyrgyzstan	WHO European Observatory
11	Lim et al. (23)	Public health communication challenges in Eastern Europe and Central Asia	Int J Environ Res Public Health, 23(1), 19	Central Asia	10.3390/ijerph23010019
12	Liu et al. (16)	Water pollution from agriculture and mining in Central Asia	Environ Pollut, 291, 118,209	Central Asia	10.1016/j.envpol.2021.118209
13	Looi (25)	Health spending driving people into poverty in Europe and Central Asia	BMJ	Europe & CA	10.1136/bmj.p735
14	Rechel et al. (1)	Primary care reforms in Central Asia	Health Policy Open, 5, 100,110	Central Asia	10.1093/eurpub/ckad187
15	Semenova et al. (2)	Historical evolution of healthcare systems of post-Soviet countries	Heliyon, 10(8), e29550	Post-Soviet	10.1016/j.heliyon.2024.e29550
16	Shaltynov et al. (14)	Inequality in distribution of healthcare resources in Kazakhstan	Int J Environ Res Public Health, 22(11), 1762	Kazakhstan	10.3390/ijerph22111762
17	Subramanian et al. (13)	Health risk in urbanizing regions: Tashkent Province	Int J Environ Res Public Health, 15(11), 2,578	Uzbekistan	10.3390/ijerph15112578

### 3.1 Overview of included studies

The 17 included studies represented a range of document types (Table 4). Peer-reviewed original research articles constituted the largest proportion (*n* = 8; 47%), followed by review and scoping review papers (*n* = 5; 29%), health system reviews and institutional reports (*n* = 2; 12%), and commentaries or editorials (*n* = 2; 12%). In terms of geographic focus, four studies examined Central Asia as a multi-country region, four focused specifically on Kazakhstan, two on Uzbekistan, one on Kyrgyzstan, one on Tajikistan, one on Iran as a comparative case, and four addressed global health diplomacy frameworks with relevance to the Central Asian context. No study focused exclusively on Turkmenistan, although it was covered within multi-country analyses.

The strongest thematic concentration was observed in health system reform and UHC (*n* = 5), followed by health diplomacy and governance (*n* = 4), environmental health (*n* = 3), healthcare inequality (*n* = 3), public health communication (*n* = 2), NCD risk factors (*n* = 2), and one study each on communicable disease control, access to medicines, and health diplomacy training. The following sections present the findings organized first by country and then by international engagement.

### 3.2 Regional public health challenges in central Asian countries

This section examines the evidence on public health diplomacy within each of the five Central Asian republics. The analysis is structured country by country to highlight the distinct health governance trajectories, domestic health challenges, and diplomatic engagement patterns that characterize each nation’s approach to health cooperation (Table 5).

**Table 5:** Country-level health diplomacy: regional role and international contribution.

Country	Key PHD challenges	Regional role	International contribution	Influence of diplomacy	SDG 3 alignment
Kazakhstan	Urban–rural healthcare inequality; declining rural facilities (35); environmental pollution from mining (36); NCD risk factors (37); uneven resource distribution (14)	Hosts WHO Country Office for CA(Central Asia) sub-region; venue for international health conferences; leads regional policy dialog on UHC; most active research producer in the region	Participates in multilateral governance (WHO, World Bank); Belt and Road Initiative (BRI) engagement through SCO; EAEU pharmaceutical harmonization; bilateral health agreements with EU member states (4)	International engagement has supported UHC policy dialog, health-system reform, technical cooperation, and pharmaceutical regulatory harmonization	SDG 3.8 (UHC equity); SDG 3.4 (NCDs); SDG 3.9 (pollution)
Kyrgyzstan	69% insurance but 46.3% Out of Pocket (OOP) spending (Kyrgyzstan Health Sysyten Review); health workforce maldistribution; COVID-19 exposed system vulnerabilities; rural access gaps	Community-based governance models as regional case study; open political environment enabling civil society health engagement; Eurasian Economic Union (EAEU) member for cross-border patient mobility	Recipient of World Bank, WHO, bilateral donor support (1); Central Asia Regional Economic Cooperation (CAREC)/ Asian Development Bank (ADB)health cooperation participant; SCO member; WHO IHR implementation	International assistance has supported health financing and system reforms, while regional mechanisms provide opportunities for cross-border cooperation and knowledge exchange	SDG 3.8 (UHC financial protection); SDG 3.d (health governance)
Tajikistan	2nd lowest per capita health spending in WHO-EUR (Tajikistan HSR); physician shortage in rural areas; basic benefits package ended 2023; cross-border TB from Afghanistan; 63.5% OOP spending	UHC by 2030 commitment provides regional advocacy platform; geographic gateway for cross-border disease surveillance (Afghanistan); One Health Framework participant	Recipient of multilateral development assistance (WHO, World Bank, ADB); Aga Khan Development Network operations since 1993 (12); CASI cross-border nutrition program	Development partnerships provide financial and technical support for health-system strengthening, UHC advancement, and cross-border disease control	SDG 3.8 (UHC by 2030); SDG 3.3 (communicable disease); SDG 3.d (capacity)
Turkmenistan	CRITICAL GAP: No country-specific study; restrictive information environment; under-reporting of disease burden (Semenova et al.); limited research engagement; health data not publicly accessible	Strategically positioned (borders all CA states + Iran/Afghanistan); highest per capita health spending ($581) but allocation unclear; One Health Framework signatory (2023); CIS/SCO member	Limited transparency constrains international cooperation; natural gas revenues provide potential fiscal capacity; engagement through SCO and CIS institutional frameworks	Limited evidence prevents assessment of the health-system impact of diplomatic engagement, highlighting an important research and governance gap	Unable to assess due to evidence gap
Uzbekistan	TB care transition challenges with financing misalignment (10); environmental/WASH-related disease in Tashkent Province (13); Aral Sea health legacy	Post-2016 diplomatic opening; expanding WHO program participation; CAREC/ADB engagement; regional cooperation through SCO and CIS	Expanded bilateral health agreements; BRI/Health Silk Road participation (26); increased receptivity to international technical assistance; One Health Framework participant	Growing international engagement creates opportunities for technical assistance, disease control, environmental health cooperation, and health-system reform	SDG 3.3 (TB control); SDG 3.8 (UHC); SDG 3.9 (WASH)

### 3.3 Current landscape of regional health diplomacy in Central Asia

Beyond the specific health challenges documented in the included studies, Kazakhstan has positioned itself as a regional leader in health diplomacy through several mechanisms. The country hosts the WHO Country Office for Kazakhstan, which also coordinates technical support for the broader Central Asian sub-region (1, 3).

Kyrgyzstan’s health system challenges present both needs and opportunities for public health diplomacy. The country’s active engagement with civil society and international partners provides opportunities for collaboration on health system strengthening and reform. Kyrgyzstan has received substantial international health development assistance, particularly from the World Bank, WHO, and bilateral donors, which has influenced the trajectory of health reform (1, 40). However, the sustainability of externally supported reforms and the alignment of donor priorities with national health needs remain ongoing concerns that require diplomatic negotiation and strategic engagement (1). The country’s experience with community-based health governance models could also serve as a valuable case for regional knowledge exchange through diplomatic channels.

Despite these challenges, the Tajik government has formally committed to achieving UHC by 2030, signaling political that could be leveraged through diplomatic engagement and international cooperation (9). This commitment creates a framework for development partners, regional organizations, and multilateral agencies to support Tajikistan’s health system strengthening efforts through coordinated technical assistance, capacity building, and financial support (1, 9). The country’s geographic vulnerability to natural disasters and cross-border health threats, including drug-resistant tuberculosis from neighboring Afghanistan, further underscores the importance of health diplomacy in addressing Tajikistan’s health security needs (9, 10).

#### 3.3.1 Role of non-state actors: the Aga khan development network

Non-state actors also play an important role in Tajikistan’s health landscape. The Aga Khan Development Network (AKDN), through its constituent agencies the Aga Khan Foundation (AKF) and the Aga Khan Health Services (AKHS), has operated in Tajikistan since 1993, providing 650,000 individuals with improved primary healthcare access through the capacity building of 668 doctors and nurses and the construction or rehabilitation of 273 healthcare facilities (11). AKDN’s cross-border programming, including the Central Asia Stunting Initiative (CASI) spanning Tajikistan, Pakistan, and Afghanistan, and the Aga Khan Medical Centre in Khorog with telemedicine links to university hospitals in Kabul and Karachi, illustrates how non-state actors contribute to health diplomacy objectives beyond traditional government-to-government channels (12). The inclusion of such actors broadens the conceptual framework of public health diplomacy to recognize the diverse ecology of organizations shaping health governance in Central Asia.

The under-representation of Turkmenistan in the public health diplomacy literature (n = 0) has significant implications for regional health governance. As a geographically central country sharing borders with all other Central Asian states as well as Iran and Afghanistan, Turkmenistan occupies a strategically important position for cross-border disease surveillance, environmental health cooperation, and regional health security (2). The country’s substantial natural gas revenues theoretically provide fiscal capacity for health system investment, yet the extent to which these resources are allocated to health remains unclear from available evidence (2). Engaging Turkmenistan more effectively in regional health diplomacy represents a critical priority for strengthening the Central Asian health governance architecture.

Since the political transition in 2016, Uzbekistan has pursued a more open foreign policy and has actively engaged with international organizations, regional cooperation platforms, and development partners. This diplomatic opening has created new opportunities for public health cooperation, including increased participation in WHO programs, expanded bilateral health agreements, and greater receptivity to international technical assistance (10, 13). The included literature, however, did not systematically analyze how this political transition has affected health governance or health diplomacy outcomes, representing an important area for future research (10, 13).

### 3.4 The role of public health diplomacy in strengthening regional health governance in Central Asia

Public health diplomacy (PHD) has significant potential to strengthen health governance and regional cooperation across Central Asia by facilitating collaboration among governments, international organizations, civil society, and development partners. The findings suggest that each country presents distinct opportunities for diplomatic engagement. Kazakhstan’s leadership role and established partnerships with international organizations position it as a regional hub for health diplomacy, capable of fostering knowledge exchange, technical cooperation, and coordinated responses to shared public health challenges (1, 3, 14). In Kyrgyzstan, PHD can support the alignment of donor investments with national health priorities, promote sustainable health reforms, and facilitate the dissemination of successful community-based governance models throughout the region (1). Similarly, Tajikistan’s commitment to Universal Health Coverage (UHC) provides a foundation for diplomatic engagement aimed at mobilizing technical expertise, financial resources, and regional partnerships to strengthen health systems and address cross-border threats such as drug-resistant tuberculosis and emerging infectious diseases (1, 9).

At the regional level, public health diplomacy can serve as a mechanism for enhancing collective health security, particularly in addressing transboundary health challenges that no single country can manage independently. Greater diplomatic engagement with Turkmenistan could strengthen regional disease surveillance, emergency preparedness, environmental health initiatives, and information-sharing mechanisms (2, 15), while Uzbekistan’s increasing openness to international cooperation presents opportunities for expanding bilateral and multilateral health partnerships (10, 13). Through regional platforms, international organizations, and cross-border agreements, PHD can facilitate policy harmonization, workforce development, capacity building, and coordinated action toward Sustainable Development Goal 3 (SDG 3) and Universal Health Coverage. As Central Asian countries continue to navigate evolving health, political, and environmental challenges, public health diplomacy offers a strategic framework for translating shared health priorities into sustainable regional action and stronger health governance systems.

### 3.5 Cross-regional health diplomacy themes

Beyond country-specific findings, the included literature identified several health diplomacy themes that cut across Central Asian countries and require regional or sub-regional coordination.

#### 3.5.1 Post-soviet health system transition

Multiple studies examined the common trajectory of post-Soviet health system evolution across Central Asia. Research documenting the historical evolution of healthcare systems across nine post-Soviet countries, including all five Central Asian states, found that while life expectancy has gradually recovered to pre-breakup levels, the transition from centralized Semashko models to more pluralistic health systems has been uneven and incomplete (2). All five countries continue to grapple with the legacy of hospital-centric service delivery, physician training misaligned with primary care needs, and governance structures that inadequately separate purchaser and provider functions (1–3). This shared historical legacy creates both common challenges and opportunities for regional health diplomacy, as countries can learn from each other’s reform experiences through structured policy dialog and knowledge exchange.

#### 3.5.2 Environmental health and transboundary cooperation

Environmental health emerged as a critical domain requiring transboundary public health diplomacy in Central Asia. A comprehensive review of water pollution from agriculture and mining documented widespread contamination across all five countries due to intensive irrigation practices, mining activities, and legacy environmental degradation (16). Nitrogen and phosphorus pollution from agricultural runoff was identified in irrigated zones throughout the region, while heavy metal contamination associated with the Asian Gold Belt posed direct health risks in mining communities (16). The Aral Sea catastrophe, one of the most severe environmental disasters in human history, continues to affect the health of populations in Kazakhstan and Uzbekistan through dust storms carrying contaminated sediments, respiratory disease, and water scarcity (16, 17).

The review emphasized the urgent need for joint transboundary water quality monitoring systems and coordinated environmental health governance, highlighting how environmental health diplomacy involving coordinated action among national governments, international agencies, and scientific institutions, is essential to address shared threats that do not respect national borders. Similarly, a global geospatial study documented that significant proportions of Central Asian populations continue to rely on solid fuels for cooking, contributing to household air pollution and child mortality, a challenge that requires both domestic policy action and international support for clean energy transitions (18).

#### 3.5.3 One health approach in Central Asia

The One Health approach has gained significant traction in Central Asia as a framework for integrating human, animal, and environmental health governance. The World Health Organization, in collaboration with Food and Agriculture Organization (FAO), World Organization for Animal Health (WOAH), and United Nations Environment Program (UNEP) (the Quadripartite Alliance), has actively promoted One Health activities across the region, identifying priority zoonotic diseases including brucellosis, rabies, and echinococcosis (19, 20). In November 2023, all five Central Asian governments approved the Central Asia One Health Framework for Action, facilitated by the World Bank, establishing regional cooperation on pandemic prevention, zoonotic disease surveillance, and environmental health (21). The subsequent launch of a regional One Health program in Dushanbe in 2025, bringing together over 200 participants from government agencies, scientific institutions, and international organizations, further demonstrates the potential of this framework as a platform for sustained public health diplomacy in the region (22).

From a public health diplomacy perspective, the One Health approach offers several advantages for Central Asia. It provides a scientifically grounded rationale for cross-border cooperation that transcends purely health-sector concerns, engaging agriculture, veterinary, and environmental ministries in coordinated action. It aligns with existing institutional platforms, particularly the SCO and CIS, which have mandates spanning multiple sectors relevant to One Health. Moreover, One Health implementation requires exactly the kinds of competencies that public health diplomacy develops: negotiation, consensus-building, cross-cultural communication, and evidence-based advocacy across diverse stakeholder groups (7, 19).

#### 3.5.4 Public health communication across Central Asia

Health communication was identified as a critical but underdeveloped dimension of public health diplomacy across the region. A scoping review of communication challenges in Eastern Europe and Central Asia found that centralized, top-down communication models inherited from Soviet-era governance remain dominant in all Central Asian countries. These models hinder transparency, responsiveness, and public trust in health information (23). The same review documented instances where misinformation originated from government sources, and called for more inclusive, trust-oriented communication strategies. A complementary global review found that disinformation is a systemic governance challenge rather than merely a technical problem, and that health diplomacy can play a constructive role in strengthening public engagement and trust in health information systems (24). These findings are particularly relevant to Central Asia, where low media independence and limited civil society engagement in health governance pose ongoing challenges to effective crisis communication and pandemic preparedness.

#### 3.5.5 Healthcare financing and catastrophic spending

The financial dimension of health access emerged as a cross-cutting concern across Central Asia. Analysis of WHO data across 40 countries in Europe and Central Asia revealed that catastrophic health spending is rising, with the poorest households disproportionately affected (25). In some Central Asian countries, up to 12% of households experience catastrophic health expenditure (25). The analysis concluded that targeted policy adjustments such as expanding exemption mechanisms, reducing co-payments for essential services, and increasing public health financing could significantly improve financial protection. These findings underscore the role of health diplomacy in facilitating cross-country policy learning, promoting evidence-based health financing reforms, and coordinating donor support for sustainable health financing transitions.

#### 3.5.6 The role of public health diplomacy in addressing cross-regional health challenges

The cross-regional themes identified in this review highlight the critical role that public health diplomacy can play in addressing shared health challenges across Central Asia (Table 6). The common legacy of post-Soviet health system transitions creates opportunities for regional policy dialog, technical cooperation, and mutual learning among countries facing similar governance, workforce, and health system reform challenges. Public health diplomacy can facilitate the exchange of best practices, strengthen institutional partnerships, and support the harmonization of health policies and standards across the region.

**Table 6:** Cross-regional public health diplomacy themes in Central Asia.

Theme	Country policy	Regional policy focus	Health system challenges	Standards/Indices
Post-Soviet health transition	KZ: Social health insurance (2020); KG: Family medicine model; TJ: UHC by 2030; TM: Centralized state model; UZ: Post-2016 reform opening	CIS framework for mutual recognition of medical qualifications; EAEU pharmaceutical harmonization; WHO European Region technical support	Hospital-centric delivery; physician training misaligned with primary care; inadequate purchaser-provider separation; fragmented governance (1, 2)	UHC Index (2023): KZ = 83, KG = 74, TJ = 72, TM = 81, UZ = 79 (30)
Environmental health and transboundary cooperation	National environmental agencies in each country; water quality monitoring (fragmented); mining regulation varying by country	Aral Sea Basin joint monitoring (IFAS); SCO environmental cooperation; Central Asia One Health Framework (2023) (21)	Transboundary water pollution (agriculture + mining); Aral Sea dust/respiratory disease; heavy metals in Gold Belt; household air pollution (16, 17)	SDG 6 (Clean Water); SDG 3.9 (Pollution); WHO air quality guidelines; National environmental monitoring data limited; UNEP regional reports available
Health communication and trust	Centralized, top-down models in all 5 countries; government-controlled media; limited civil society engagement in health messaging	No regional communication coordination mechanism identified; WHO crisis communication guidelines adopted unevenly	Misinformation from government sources; low media independence; public distrust in health information; weak pandemic communication (23, 24)	WHO IHR communication capacity scores; press freedom indices; WHO IHR SPAR scores: KZ = 73, KG = 55, TJ = 44, TM = N/A, UZ = 58 (30)
Health financing and catastrophic spending	KZ: 3.0% GDP on health; KG: 46.3% OOP; TJ: 63.5% OOP (2nd lowest in WHO-EUR); TM: highest per capita but limited transparency; UZ: expanding public spending post-2016	WHO/World Bank joint monitoring of UHC financial protection; ADB CAREC health financing cooperation; EU technical assistance for health financing reform	Rising catastrophic spending (up to 12% of households); poorest disproportionately affected; medications outside benefits packages; co-payments barriers (14, 25)	SDG 3.8.2 (catastrophic spending); Health expenditure per capita (2023): KZ = $490, KG = $97, TJ = $85, TM = $581, UZ = $192 (21)
One health and zoonotic diseases	National One Health focal points established; joint human-animal surveillance in KZ, KG, TJ; veterinary-public health coordination developing	Central Asia One Health Framework for Action (2023) approved by all 5 countries; Quadripartite Alliance (WHO/FAO/WOAH/UNEP) support; regional program launched (22)	High burden of brucellosis, rabies, echinococcosis; zoonotic spillover risk; agricultural economies increase human-animal interface (19, 21)	WOAH performance indicators; FAO antimicrobial surveillance; WHO Joint External Evaluation (JEE) scores

WHO (30); World Bank (21); Rechel et al. (1); Semenova et al. (2); Liu et al. (16); Lim et al. (23); Looi (25); Kasymbekova et al. (19). KZ, Kazakhstan; KG, Kyrgyzstan; TJ, Tajikistan; TM, Turkmenistan; UZ, Uzbekistan.

Similarly, environmental health threats, health misinformation, and financial barriers to healthcare access transcend national borders and require coordinated responses that extend beyond traditional public health interventions. Public health diplomacy provides a mechanism for fostering transboundary cooperation on environmental monitoring, disease surveillance, risk communication, and health financing reforms through engagement among governments, international organizations, academic institutions, and civil society actors. By promoting collaborative governance, resource mobilization, and knowledge sharing, public health diplomacy can strengthen regional health security, advance progress toward Universal Health Coverage (UHC), and support the achievement of Sustainable Development Goal 3 (SDG 3) across Central Asia. These findings suggest that health challenges in the region are not solely national concerns but shared regional issues that require sustained diplomatic engagement and multisectoral collaboration.

### 3.6 International health diplomacy with Central Asia

This section examines the evidence on how international actors including regional organizations, bilateral partners, and multilateral institutions engage in different health diplomacy with Central Asian countries. The analysis considers both the mechanisms and the effectiveness of international health cooperation in the region (Table 7).

**Table 7:** International health diplomacy engagement with Central Asia: Mechanisms, findings, and indicators.

International actor	Actor type	Health diplomacy mechanisms	Key findings	Numerical indicators	Evidence gaps
European Union	Regional Political and Economic Union	EU-Central Asia Strategy; development cooperation; health system strengthening; academic partnerships; regulatory harmonization	Health used as soft-power diplomatic tool; effectiveness depends on genuine health outcomes vs. symbolic cooperation (4)	EU development aid to CA: €750 M (2014–2020 Multiannual Financial Framework/MFF); EU-CA health programs: 12 + active projects (2018–2024); Erasmus+ partnerships: 40 + university links (European Commission, 2024)	Limited impact evaluation of EU health programs in CA
China/BRI/SCO	Bilateral partner and regional organization	Belt and Road Initiative (Health Silk Road); SCO health cooperation; vaccine diplomacy; hospital construction; pharmaceutical supply	Significant investment for health infrastructure; complex geopolitical navigation; SCO health frameworks under-utilized (Zhang (29);)	BRI health infrastructure: $2.2B committed to CA (2015–2023); SCO member states: 10 (incl. 5 CA); COVID-19 vaccines supplied by China to CA: ~50 M doses (2020–2022) (41)	No empirical evidence on SCO health cooperation outcomes
Russia/CIS/EAEU	Bilateral Partner & Regional Economic Union	Soviet institutional legacy; CIS health cooperation; EAEU pharmaceutical regulation; migrant health governance	Historical influence on health governance; migrant health under-examined; cross-border patient mobility frameworks exist (Semenova et al.)	CA labor migrants in Russia: est. 3–5 million; CIS member states: 9 (all 5 CA); EAEU members: KZ, KG (CA); pharmaceutical mutual recognition agreements	Migrant health diplomacy largely absent from literature
WHO	Multilateral Organization (UN Agency)	IHR coordination; health system reform support; country office technical assistance; Global Competency Framework for UHC	Key institutional framework; translates global norms to national contexts; capacity building support (7, 30)	UHC Index range: 72–83 (2023); WHO country offices: 5 (all CA states); IHR SPAR avg. scores: KZ = 73, KG = 55, TJ = 44, TM = N/A, UZ = 58 (30); JEE completed: KZ (2017), KG (2016)	Adoption of WHO frameworks in CA not systematically documented
World Bank	International Financial Institution	Health financing reform; results-based financing; policy lending; hospital rationalization; analytical work (PERs, HSPAs)	Significant influence on reform design in KZ, KG, TJ; sustainability concerns after project completion (1)	Active health projects in CA: 8 (2024); total health lending to CA: ~$1.5B cumulative; countries with active WB health support: KZ, KG, TJ (21)	Alignment of Bank priorities with national health needs
ADB/CAREC	International Financial Institution	CAREC 2030 health cooperation; health sector approach; cross-border surveillance; pharmaceutical harmonization	Complements WHO/World Bank through distinct institutional channels; regional health cooperation opportunities identified (31, 32)	CAREC member countries: 11 (includes all 5 CA); ADB health sector investment in CA: ~$800 M cumulative (31)	Limited evaluation of CAREC health cooperation outcomes
AKDN/non-state	International Non-Governmental Organization	Primary healthcare capacity building; facility construction; cross-border nutrition programs (CASI); telemedicine (Khorog-Kabul-Karachi)	650,000 individuals gained healthcare access; 668 doctors/nurses trained; 273 facilities built in Tajikistan since 1993 (11)	AKDN operations: primarily TJ + cross-border with AF, PK; CASI: 3 countries; Aga Khan Medical Centre: 1 (Khorog); facilities built/rehabilitated: 273	Non-state actor health diplomacy underrepresented in literature
Global frameworks	Multilateral Governance Frameworks	GHD governance; NCD diplomacy; access to medicines; health diplomacy training	COVID-19 exposed multilateral weaknesses; 2 billion lack medicine access; no CA-specific training programs (27, 34)	Global essential medicines access gap: 2 billion people; GHD training programs globally: 8 identified; CA-specific programs: 0	Applicability of global frameworks to CA context not tested

#### 3.6.1 European Union–Central Asia health diplomacy

The most direct evidence of international health diplomacy in Central Asia came from a case study examining health cooperation between the European Union and the region. The study found that the EU increasingly deploys health as a diplomatic instrument within its broader foreign policy toward Central Asia, with health initiatives serving dual purposes: improving population health outcomes and advancing strategic diplomatic relationships (4). EU-funded health programs in the region have focused on areas including maternal and child health, communicable disease control, health system strengthening, and regulatory harmonization.

However, the study raised important questions about the accountability and impact measurement of health diplomacy initiatives. The effectiveness of EU health diplomacy was found to depend critically on whether improved health outcomes are genuinely achieved or whether health cooperation remains primarily symbolic serving diplomatic signaling purposes without producing measurable health gains (4). This finding highlights a fundamental tension in health diplomacy: the dual mandate of serving both health objectives and foreign policy interests can create misalignment when diplomatic priorities diverge from population health needs.

The EU’s health diplomacy in Central Asia also operates through institutional frameworks including the EU-Central Asia Strategy, which explicitly identifies health as an area of cooperation. The European Commission’s development cooperation instruments have supported health system strengthening projects across the region, while European academic institutions have partnered with Central Asian universities on health research capacity building (4). These engagements represent a multifaceted approach to health diplomacy that extends beyond traditional government-to-government negotiations to encompass institutional, academic, and civil society dimensions.

#### 3.6.2 China, the belt and road initiative, and the Shanghai cooperation organization

China’s growing engagement in Central Asia through the Belt and Road Initiative (BRI) and the Shanghai Cooperation organization (SCO) was identified as a significant dimension of international health diplomacy in the region. Research on China’s development strategy in Central Asia documented that Chinese investment and cooperation activities have substantial implications for health infrastructure, including hospital construction, medical equipment provision, and pharmaceutical supply chains (26). The BRI’s health component, sometimes referred to as the “Health Silk Road”, has expanded since the COVID-19 pandemic, with China providing vaccines, medical supplies, and public health technical assistance to Central Asian partner countries (26, 27).

China’s health engagement in Central Asia serves multiple strategic objectives beyond humanitarian concern: expanding geopolitical influence in a region historically dominated by Russia, securing access to Central Asian energy resources and trade corridors (26), building diplomatic goodwill to support broader Belt and Road Initiative investments (26, 27), and positioning China as an alternative development partner to Western institutions (4, 27). This strategic dimension means that health cooperation outcomes in the region are shaped not only by public health needs but also by China’s broader foreign policy calculations (27), underscoring the importance of Central Asian countries developing the diplomatic capacity to negotiate health partnerships that prioritize national health priorities over external geopolitical agendas (28, 29).

However, the effectiveness of China’s health engagement depends on careful consideration of geopolitical and geoeconomic factors. Changing political dynamics among Central Asian states and between Central Asia and major external powers including Russia, China, the United States, and the EU, create a complex diplomatic environment in which health cooperation must be navigated. The SCO, which includes all five Central Asian states as well as China, Russia, India, and Pakistan, has established health cooperation mechanisms including joint disease surveillance agreements and public health emergency response coordination (26, 29). Yet the included literature did not provide empirical evidence on the implementation or outcomes of these SCO health cooperation frameworks, suggesting that the institutional potential for health diplomacy through this platform remains under-utilized and under-studied (26, 29).

#### 3.6.3 Russia and the commonwealth of independent states

Although Russia’s role as a health diplomacy actor in Central Asia was not the primary focus of any included study, its influence was acknowledged across several analyses. Russia’s historical legacy as the metropolitan center of the Soviet health system continues to shape Central Asian health governance through institutional models, medical education standards, and pharmaceutical regulatory frameworks that bear the imprint of Soviet-era practices (2). The Commonwealth of Independent States (CIS) and the Eurasian Economic Union (EAEU) provide institutional platforms for health cooperation among post-Soviet states, including provisions for cross-border patient mobility, mutual recognition of medical qualifications, and coordinated pharmaceutical regulation (1, 2).

The included literature identified the substantial flows of labor migrants between Central Asian countries and Russia as a significant but under-examined dimension of health diplomacy. Millions of Central Asian citizens work in Russia, where they face challenges in accessing health services, are exposed to occupational health risks, and may serve as vectors for cross-border disease transmission (1). The governance of migrant health including bilateral agreements on health service access, disease surveillance coordination, and social protection represents an important opportunity of public health diplomacy between Russia and Central Asian states.

#### 3.6.4 WHO and multilateral health governance

The World Health Organization’s engagement in Central Asia was referenced across multiple included studies as a key institutional framework for health diplomacy. The WHO Regional Office for Europe, under which the Central Asian countries fall, provides technical support for health system reforms, coordinates disease surveillance through the International Health Regulations, and facilitates capacity building for health governance (30). WHO’s country offices in Central Asia serve as critical nodes for translating global health norms and standards into national policy contexts, and for supporting government engagement in multilateral health governance processes (30).

The Global Competency Framework for Universal Health Coverage, developed by WHO, was identified in the included literature as a relevant framework for health diplomacy training and workforce development in Central Asia (7, 30). The framework articulates six domains of health worker competencies: people-centeredness, decision-making, communication, collaboration, evidence-informed practice, and personal conduct. These competencies can be directly applicable to the practice of public health diplomacy (7, 30). However, the extent to which Central Asian health systems have adopted or adapted this framework was not systematically documented in the included studies.

#### 3.6.5 World Bank and development finance for health

The World Bank’s role in shaping health system reform in Central Asia was evident across several studies included, particularly those examining health financing, primary care reform, and UHC progress. World Bank analytical work including Public Expenditure Reviews, Health System Performance Assessments, and policy lending operations has significantly influenced the design and implementation of health reforms in Kazakhstan, Kyrgyzstan, and Tajikistan (1, 9). The Bank’s technical assistance has supported the introduction of results-based financing mechanisms, health insurance reforms, and hospital rationalization programs across the region.

From a health diplomacy perspective, the World Bank’s engagement illustrates both the potential and the limitations of international financial institution involvement in health governance. While Bank-supported reforms have contributed to strengthening health financing and service delivery in several countries, questions remain about the sustainability of reforms after project completion, the alignment of Bank priorities with national health needs, and the extent to which conditionality attached to lending operations constrains domestic policy space for health governance innovation.

#### 3.6.6 Asian development bank and regional health cooperation

In addition to the World Bank, the Asian Development Bank (ADB) represents a significant actor in health-related development finance and diplomacy in Central Asia. ADB’s Central and West Asia health sector approach outlines targeted strategies for supporting developing member countries in the health sector, with a focus on strengthening primary healthcare, expanding health insurance coverage, and improving health infrastructure across the region (31). Through the Central Asia Regional Economic Cooperation (CAREC) program, ADB has supported regional health cooperation initiatives that align directly with public health diplomacy objectives, including cross-border disease surveillance, health system harmonization, and coordinated responses to shared health threats (32).

The CAREC 2030 regional health cooperation study identified opportunities for deepening health cooperation among member countries, including harmonization of pharmaceutical regulation, joint health workforce development, and collaborative approaches to pandemic preparedness and response (32). These initiatives complement WHO and World Bank activities while operating through distinct institutional channels and governance mechanisms, creating a layered architecture of international health cooperation in Central Asia. From a public health diplomacy perspective, ADB’s engagement illustrates the importance of recognizing the full range of multilateral development actors shaping health governance in the region.

#### 3.6.7 Global health diplomacy frameworks and their applicability to Central Asia

Several studies provided global health diplomacy frameworks with potential applicability to the Central Asian context. A Lancet series paper on global health diplomacy examined how the COVID-19 pandemic exposed weaknesses in multilateral health governance at a time when international cooperation was already under geopolitical strain (27). The analysis argued that global health has become integral to geopolitics, and that new international relations concepts are needed to capture the complexities of health diplomacy in an era of great power competition. These insights are directly relevant to Central Asia, where competing influences from Russia, China, the EU, and the United States create a multipolar diplomatic environment that shapes the possibilities and constraints for health cooperation (27).

Research on health diplomacy in Iran provided a comparative perspective from another post-revolutionary state navigating complex international relationships. The study found that a lack of consensus on the definition and scope of global health diplomacy among Iranian policymakers led to reactive, crisis-driven approaches rather than systematic integration of health into foreign policy (33). This finding resonates with the Central Asian context, where health diplomacy has often been driven by external actors and emergency responses such as pandemic preparedness and disease outbreak management rather than by proactive, nationally owned strategies for leveraging health cooperation to advance broader development and security objectives (33, 38).

A global review on access to medicines through health diplomacy documented that approximately 2 billion people worldwide lack access to essential medicines, with access gaps disproportionately affecting Low- and Middle-Income Countries (LMICs) including Central Asian countries (34). The review argued that global health diplomacy contributes to addressing treatment disparities through multilateral negotiations on intellectual property, pricing mechanisms, and supply chain cooperation. Central Asian countries could benefit from more active participation in international pharmaceutical diplomacy, regional procurement cooperation, and engagement with initiatives such as the Medicines Patent Pool and Trade-Related Aspects of Intellectual Property Rights (TRIPS) flexibilities (34).

#### 3.6.8 Health diplomacy training and capacity building

The capacity of Central Asian health professionals and policymakers to engage effectively in health diplomacy was addressed through a global scoping review of health diplomacy training initiatives. The review identified eight educational programs and frameworks globally, emphasizing negotiation, cross-cultural communication, leadership, and policy analysis as core competencies (7). However, no training program originating from or specifically tailored to the Central Asian context was identified, representing a significant capacity gap. The absence of locally relevant health diplomacy education may limit the ability of Central Asian professionals to effectively represent their countries’ health interests in multilateral negotiations, to navigate the complex geopolitical dynamics of health cooperation in the region, and to build sustainable communities of practice for public health diplomacy.

#### 3.6.9 The role of public health diplomacy in international health cooperation

The findings demonstrate that public health diplomacy provides a critical mechanism for translating international partnerships, development assistance, and multilateral engagement into tangible health gains across Central Asia. The diverse involvement of external actors, including the European Union, China, Russia, the World Health Organization, and the World Bank, illustrates how health has become an increasingly important component of foreign policy, regional cooperation, and development strategies. Public health diplomacy can help Central Asian countries navigate this complex geopolitical environment by ensuring that international health partnerships align with national health priorities, strengthen health systems, and contribute to long-term population health outcomes. Effective diplomatic engagement can also enhance countries’ ability to negotiate technical assistance, mobilize financial resources, facilitate technology transfer, and participate more actively in regional and global health governance processes (39).

Furthermore, public health diplomacy offers opportunities to strengthen regional collaboration on shared health challenges that transcend national borders, including communicable disease outbreaks, migrant health, environmental health threats, access to medicines, and health workforce development. The findings suggest that while numerous international health cooperation mechanisms exist, their effectiveness depends on local ownership, institutional capacity, and sustained diplomatic engagement. Strengthening public health diplomacy competencies among policymakers, public health professionals, and academic institutions could improve Central Asian countries’ ability to shape international health agendas, advocate for regional priorities, and build equitable partnerships with external actors. As the region continues to navigate competing geopolitical influences and evolving public health challenges, public health diplomacy can serve as a strategic tool for advancing health governance, regional health security, Universal Health Coverage (UHC), and Sustainable Development Goal 3 (SDG 3).

### 3.7 Alignment of identifies themes with SDG 3 and universal health coverage

The alignment of included studies with specific SDG 3 targets varied across the literature. The strongest alignment was observed with SDG 3.8 (universal health coverage), which was directly addressed by studies examining health system reforms, primary care strengthening, health financing, and access to medicines (*n* = 8) (1, 14, 34). SDG 3.4 (reduction of premature non Communicable disease (NCD) mortality) was addressed by studies on dietary patterns, industrial pollution health impacts, and NCD diplomacy frameworks (*n* = 3). SDG 3.3 (communicable disease control) was addressed through research on TB treatment and infectious disease burden (*n* = 2). SDG 3.d (strengthening health governance capacity) was indirectly addressed by studies on health communication, crisis preparedness, and health diplomacy training (*n* = 3).

Several important SDG 3 targets received limited or no direct attention through a health diplomacy lens. SDG 3.1 (maternal mortality reduction), SDG 3.2 (ending preventable child deaths), SDG 3.5 (substance abuse prevention), and SDG 3.a (tobacco control) were not explicitly examined in the Central Asian health diplomacy literature. This gap suggests that the regional health diplomacy discourse has been dominated by health system and governance concerns, with insufficient attention to specific disease and population health targets critical to the overall SDG 3 agenda. This is also an opportunity to facilitate public health diplomacy through advance local, regional and global cooperation and collaboration.

### 3.8 Assessment of gaps in literature

#### 3.8.1 Geographic gaps

The most striking geographic gap is the complete absence of country-specific evidence on Turkmenistan. Tajikistan is similarly under-represented with only one health system review. The concentration of research on Kazakhstan, while reflecting its larger academic infrastructure, means that the health diplomacy challenges of the most resource-constrained countries remain inadequately documented. No study examined the five Central Asian countries comparatively through a public health diplomacy analytical framework, which would enable systematic identification of shared challenges and differentiated policy responses.

#### 3.8.2 Institutional and organizational gaps

The role of regional organizations, particularly the SCO, CIS, and EAEU in facilitating health cooperation received minimal empirical attention despite these organizations’ institutional mandates and platforms for health governance. The diplomatic practices, negotiation processes, and institutional mechanisms through which health cooperation is operationalized in Central Asia remain largely undocumented. No study systematically mapped the bilateral health agreements between Central Asian countries or between Central Asian countries and external partners, which would provide a foundation for assessing the landscape of health diplomacy commitments and implementation.

#### 3.8.3 Thematic gaps

Mental health, substance abuse, reproductive health, and antimicrobial resistance, areas of significant public health importance in Central Asia were not examined through a health diplomacy lens. The impact of labor migration on cross-border health governance was notably absent despite the substantial migrant flows between Central Asia and Russia. Capacity building and education is another aspect of research interest in the region. Pandemic preparedness and health security cooperation, despite their heightened relevance following COVID-19, received limited systematic attention in the context of Central Asia health diplomacy. Climate change and its health impact, including water scarcity, extreme heat events, and food security, represent an emerging but unaddressed domain for health diplomacy in the region.

#### 3.8.4 Methodological gaps

No randomized controlled trials, quasi-experimental studies, or formal impact evaluations of health diplomacy interventions in Central Asia were identified. The predominance of descriptive and qualitative approaches means that the evidence base for effectiveness remains weak. No study measured the outcomes of diplomatic engagement on health indicators, policy adoption, or institutional capacity, making it difficult to assess the return on investment of health diplomacy activities in the region. The absence of standardized metrics for evaluating health diplomacy outcomes limits comparability across studies and countries.

## 4 Discussion

This scoping review provides the first comprehensive mapping of the evidence on health diplomacy in Central Asia. The findings reveal a nascent but critically important field shaped by the region’s post-Soviet institutional legacy, its position at the intersection of competing geopolitical influences, and its diverse but interconnected health challenges. The following discussion contextualizes these findings within the broader literature on health diplomacy, identifies implications for policy and practice, and acknowledges the limitations of this review.

### 4.1 Unevenness of the evidence base across Central Asia

The most striking finding is the profound unevenness of the evidence base across Central Asian countries. Kazakhstan dominates the literature, reflecting its larger economy, more developed academic infrastructure, and greater openness to international research collaboration. At the opposite extreme, Turkmenistan is virtually absent, a gap that reflects the country’s restrictive information environment but also represents a critical blind spot for regional health governance. This unevenness has practical consequences: health diplomacy strategies for the region cannot be evidence-informed if the evidence is systematically missing for the countries where health governance challenges may be most acute.

The country-level analyses reveal that all five Central Asian republics continue to navigate the complex post-Soviet health transition, but at markedly different paces and with distinct institutional configurations. Kazakhstan’s introduction of mandatory social health insurance represents the most ambitious UHC reform in the region, yet the persistence of urban–rural disparities and declining rural facility density demonstrate that structural reform alone is insufficient without sustained attention to equitable resource distribution (14, 35). Kyrgyzstan’s relatively broad insurance coverage (69%) is undermined by catastrophic out-of-pocket spending that pushes households into poverty. Tajikistan’s UHC commitment by 2030, while politically significant, faces formidable structural barriers given its extremely constrained fiscal environment. Uzbekistan’s post-2016 diplomatic opening has created new possibilities for health cooperation, but the evidence on whether this political transition has produced measurable health governance improvements remains limited.

These findings align with the broader global health diplomacy literature, which has documented similar patterns of uneven evidence generation across low- and middle-income countries (27). The Central Asian case is distinctive, however, in the degree to which post-Soviet institutional legacies including centralized governance models, hospital-centric service delivery, and pharmaceutical regulatory frameworks, continue to shape health system design and constrain reform options. This shared legacy creates a foundation for regional policy dialog and peer learning that is not available to regions without comparable institutional histories.

### 4.2 International health diplomacy: opportunities and tensions

The findings on international engagement reveal a complex, multipolar diplomatic environment. The EU’s use of health as a soft-power instrument raises fundamental questions about accountability: when health cooperation serves both population health improvement and strategic diplomatic interests, whose definition of effectiveness should prevail? This tension, identified by Collins et al. (4), is not unique to the EU-Central Asia relationship but reflects a broader challenge in global health diplomacy where health objectives and foreign policy interests may diverge.

China’s expanding engagement through the Belt and Road Initiative (BRI) and Shanghai Cooperation Organization (SCO) represents perhaps the most significant shift in the health diplomacy landscape of Central Asia in recent decades. The “Health Silk Road” initiative, vaccine diplomacy during COVID-19, and infrastructure investments have created new channels for health cooperation outside traditional Western-led frameworks. However, the absence of empirical evidence on SCO health cooperation outcomes suggests that institutional potential remains underutilized. This finding echoes Pattanshetty et al.’s (29) broader observation that international relations theories have been insufficiently applied to health security, resulting in a gap between institutional frameworks and evidence-based evaluation.

Russia’s enduring influence on Central Asian health governance through institutional frameworks, medical education, and pharmaceutical regulation deserves greater analytical attention. The governance of migrant health involving millions of Central Asian workers in Russia represents a particularly urgent but neglected dimension of health diplomacy with implications for disease surveillance, social protection, and human rights. The included literature largely overlooked this dimension, despite its significance for population health in both sending and receiving countries.

Recent scholarship on health geopolitics in Central Asia highlights that health investments and technical assistance programs are not politically neutral but carry implicit assumptions about governance models, regulatory standards, and institutional designs reflecting the priorities of the providing actor (15). The concept of health sovereignty, the capacity of states to make autonomous decisions about health policy, pharmaceutical procurement, and health system design, has emerged as a relevant framework for analyzing health diplomacy in post-Soviet contexts. Central Asian countries must balance the benefits of international cooperation against dependency risks, and PHD offers a framework for navigating these tensions by empowering national actors to engage from a position of informed agency rather than passive reception (15).

The WHO and World Bank play foundational roles in shaping health reforms across the region, yet questions about sustainability, alignment with national priorities, and the constraints imposed by conditionality remain salient. The Global Competency Framework for Universal Health Coverage offers a structured approach to workforce development that is directly applicable to health diplomacy capacity building, but its adoption in Central Asia has not been systematically assessed.

### 4.3 Environmental health diplomacy

The findings on environmental health challenges including transboundary water pollution, industrial contamination, the Aral Sea catastrophe, and household air pollution reveal a dimension of public health diplomacy that has been underappreciated in the broader GHD literature. Unlike many dimensions of health diplomacy that operate through international organizations and multilateral frameworks, environmental health diplomacy in Central Asia requires direct negotiation among neighboring states over shared natural resources. The shared water systems of the Amu Darya and Syr Darya rivers, the legacy of Soviet-era environmental degradation, and the health impacts of mining and agricultural contamination create compelling imperatives for diplomatic cooperation that extend beyond traditional health sector boundaries (16). This makes environmental health diplomacy one of the most tangible and consequential arenas for public health diplomatic engagement in the region.

### 4.4 Health communication, trust, and governance

The persistence of centralized, top-down health communication models across Central Asia has significant implications for health diplomacy (23). Effective health diplomacy requires not only government-to-government negotiation but also public trust in health institutions and information systems. The COVID-19 pandemic demonstrated globally that health governance effectiveness depends on public compliance, which in turn depends on trust. In Central Asian countries where media independence is limited and civil society engagement in health governance is constrained, building transparent, trust-oriented communication systems is both a public health priority and a diplomatic challenge. The finding that misinformation sometimes originates from government sources themselves (23, 24) underscores the depth of this governance challenge.

### 4.5 Health diplomacy capacity gap

The absence of health diplomacy training programs specific to Central Asia represents a critical capacity gap. While global frameworks exist including the Global Health Diplomacy Network (GHDIN) competency model and the eight training initiatives identified by Joshi et al. (7). Their applicability to the Central Asian context has not been assessed. The region’s distinctive characteristics, post-Soviet governance traditions, multilingual populations, competing geopolitical pressures, and shared environmental challenges demand locally developed curricula that equip professionals with contextually relevant skills in negotiation, cross-cultural communication, policy analysis, and crisis management. Without such capacity, Central Asian countries risk remaining primarily recipients of externally driven health cooperation rather than active agents in shaping regional and global health governance.

### 4.6 Alignment with SDG 3

The strongest alignment between the included literature and SDG 3 was observed with target 3.8 (UHC), reflecting the centrality of health system reform and health financing to the regional health agenda. However, the absence of evidence on maternal mortality (SDG 3.1), child health (SDG 3.2), substance abuse (SDG 3.5), and tobacco control (SDG 3.a) through a health diplomacy lens represents a significant gap. These targets address health challenges of major importance in Central Asia, the region has among the highest rates of tobacco consumption globally, and maternal and child health outcomes vary dramatically between urban and rural areas, yet they have not been examined through the analytical lens of public health diplomacy. This suggests that the health diplomacy discourse in the region has been captured by system-level governance concerns at the expense of specific population health targets.

### 4.7 Strengths and limitations

This review has several strengths. It provides the first comprehensive mapping of opportunities for public health diplomacy in Central Asia, synthesizing evidence across diverse disciplines and document types. The country-by-country analytical structure enables differentiated analysis, while the thematic synthesis identifies cross-cutting issues. The inclusion of gray literature ensures broader coverage of a field where much relevant evidence exists outside traditional academic channels.

Several limitations must be acknowledged. First, the restriction to English and Russian publications may have excluded relevant literature in national languages (Kazakh, Kyrgyz, Tajik, Turkmen, Uzbek). Second, the scoping review methodology does not allow formal assessment of intervention effectiveness or study quality. Third, heterogeneity among included studies limits comparability. Fourth, the reliance on published literature underrepresents health diplomacy activities conducted through bilateral agreements, institutional memoranda, and diplomatic processes that are not documented in academic or gray literature. Fifth, the estimated numbers in the PRISMA flow diagram were derived from the compiled document provided and may differ when the full *de novo* search is executed.

### 4.8 Recommendations

Based on the findings of this review, several key recommendations emerge. There is a need to prioritize evidence generation in underrepresented countries, particularly Turkmenistan and Tajikistan, through collaborative and transparent research partnerships that strengthen health system evidence while respecting national contexts. Regional health cooperation platforms such as the Shanghai Cooperation Organization, CIS, and EAEU should be more systematically evaluated and leveraged to enhance health diplomacy mechanisms. Central Asian academic institutions should develop locally relevant health diplomacy training programs adapted to regional geopolitical and health system realities rather than relying solely on global frameworks. Greater emphasis is also needed on environmental health diplomacy to address shared transboundary challenges, alongside strengthened bilateral and multilateral approaches to migrant health governance, particularly concerning labor migration to Russia. Future research should adopt robust longitudinal and quasi-experimental designs to assess the impact of health diplomacy on health outcomes, while also broadening the SDG 3 agenda to include priority health issues such as maternal and child health, antimicrobial resistance, and non-communicable diseases, ensuring a more comprehensive and action-oriented approach to public health diplomacy in Central Asia.

## 5 Policy directions for public health diplomacy in Central Asia

The findings of this review point to four key policy directions for strengthening public health diplomacy across Central Asia.

### 5.1 National-level policy directions

Central Asian governments should formally integrate health into their foreign policy frameworks, moving beyond reactive, crisis-driven approaches toward systematic health diplomacy strategies (33). Each country should designate health diplomacy focal points within their ministries of foreign affairs and incorporate cross-border cooperation provisions into national health policies, particularly for tuberculosis control, antimicrobial resistance, and pandemic preparedness.

### 5.2 Regional-level policy directions

Existing regional platforms: the SCO, CIS, and EAEU, should be leveraged more strategically for health cooperation, as their institutional potential remains underutilized (26, 29). A dedicated Central Asian Health Diplomacy Forum, modeled on the One Health Framework for Action (21), should be established for annual ministerial-level dialog on shared health priorities. Regional pharmaceutical procurement cooperation should be pursued to improve access to essential medicines, particularly for Tajikistan and Kyrgyzstan where out-of-pocket spending creates severe barriers to care (25, 34).

### 5.3 Environmental and one health policy directions

Environmental health diplomacy should be elevated as a regional priority. Joint water quality monitoring across the Amu Darya and Syr Darya basins should incorporate health indicators linking environmental surveillance to population health outcomes (16). The One Health Framework should be expanded to include public health diplomacy competencies in its governance components (19, 21).

### 5.4 Workforce and capacity building policy directions

Central Asian universities should develop locally tailored health diplomacy curricula grounded in the region’s geopolitical context rather than relying on imported frameworks (7). The WHO Global Competency Framework for UHC should be adapted for the Central Asian context (30). Professional exchange programs between Central Asian health professionals and established health diplomacy training centers should be facilitated to build sustainable communities of practice across the region.

## 6 Conclusion

This scoping review has mapped the landscape of public health diplomacy in Central Asia, highlighting both its emerging importance and significant evidence gaps. The five Central Asian republics-Kazakhstan, Kyrgyzstan, Tajikistan, Turkmenistan, and Uzbekistan-share a common post-Soviet health system legacy but demonstrate divergent reform trajectories, creating both shared challenges and varied opportunities for regional cooperation. The findings show that public health diplomacy operates within a complex multipolar geopolitical environment involving actors such as the European Union, China, Russia, and multilateral organizations, offering both opportunities for resource mobilization and risks of fragmented health governance shaped by broader political interests.

At the country level, while notable progress has been made in health reforms and commitments to Universal Health Coverage, persistent inequalities, financing constraints, and environmental health burdens continue to hinder equitable outcomes, with the lack of evidence on Turkmenistan representing a critical regional blind spot. Environmental health challenges, particularly transboundary water pollution and the legacy of the Aral Sea disaster, further underscore the need for sustained diplomatic cooperation across sectors and borders. Additionally, the absence of Central Asia-specific health diplomacy training highlights a key capacity gap, limiting the region’s ability to actively shape global and regional health governance. Overall, strengthening public health diplomacy is essential not only for improving health system performance but also for advancing regional health security, equity, and progress toward Universal Health Coverage across Central Asia.
